# A Note on the Observational Evidence for the Existence of Event Horizons in Astrophysical Black Hole Candidates

**DOI:** 10.1155/2013/204315

**Published:** 2013-06-17

**Authors:** Cosimo Bambi

**Affiliations:** ^1^Arnold Sommerfeld Center for Theoretical Physics, Ludwig Maximilians University Munich, 80333 Munich, Germany; ^2^Department of Physics, Fudan University, Shanghai 200433, China

## Abstract

Black holes have the peculiar and intriguing property of having an event horizon, a one-way membrane causally separating their internal region from the rest of the Universe. Today, astrophysical observations provide some evidence for the existence of event horizons in astrophysical black hole candidates. In this short paper, I compare the constraint we can infer from the nonobservation of electromagnetic radiation from the putative surface of these objects with the bound coming from the ergoregion instability, pointing out the respective assumptions and limitations.

## 1. Introduction

A black hole (BH) can be defined as the region *ℬ* of the total spacetime *ℳ* which does not overlap with the causal past of future null infinity *J* ^−^(*ℐ* ^+^) [1]:

(1) $$\begin{array}{c} \;ℬ=ℳ-J^{-}\left({ℐ}^{+}\right). \end{array}$$

The *event horizon* of a BH is the boundary delimiting the BH. Everything falling onto the BH and crossing the event horizon is lost forever and it cannot affect events happening outside the BH any more. However, it may be possible that event horizons never form in nature but that only apparent horizons can be created [2–4]. An *apparent horizon* is a closed surface of zero expansion for a congruence of outgoing null geodesics orthogonal to the surface [1]. Outward-pointing light rays behind an apparent horizon actually move inwards and therefore they cannot cross the apparent horizon. In the special case of a stationary spacetime, an event horizon is also an apparent horizon, but the reverse is not true in general. In particular, the event horizon is determined by the global properties of the spacetime, while the apparent horizon depends on the observer.

Astronomers have discovered at least two classes of astrophysical BH candidates [5]: stellar-mass objects in X-ray binary systems and supermassive objects at the center of every normal galaxy. These objects are thought to be BHs because they cannot be explained otherwise without introducing new physics: the stellar-mass BH candidates are too heavy to be neutron stars for any reasonable matter equation of state [6, 7], while at least some of the supermassive objects in galactic nuclei are too heavy, compact, and old to be clusters of nonluminous bodies [8]. There is also a set of observations suggesting that BH candidates have really an event horizon [9–13]. Basically, these objects seem to be able to swallow all the accreting gas without emitting any kind of electromagnetic radiation from their putative surface. In the case of low-mass X-ray binaries, we can compare systems in which the primary is thought to be a BH and the ones in which the primary is thought to be a neutron star. In the quiescent state, we can observe thermal radiation from the surface of neutron stars, while no such a radiation is observed from BH candidates [9, 10]. Neutron star systems show type-I X-ray bursts (as outcome of compression and heating of the gas accumulated on their surface), while the phenomenon has never been observed in binaries with BH candidates [11]. There are also strong constraints on the radiation emitted by the possible surface of the supermassive BH candidate at the center of our Galaxy [12, 13].

This body of observations can be easily explained with the fact that BHs have no surface and that the gas crossing the event horizon cannot be seen by distant observers any more (see however ref. [14]). Strictly speaking, the confirmation for the existence of an event horizon would require the knowledge of the future null infinity of the Universe, which is clearly impossible for us. On the contrary, the nonobservation of electromagnetic radiation emitted by the gas after falling into the compact object nicely meets the definition of apparent horizon. However, the geometry of the spacetime around astrophysical BH candidates is practically stationary for the timescale of our observations, and that may make it impossible to discriminate an event horizon from an apparent horizon.

## 2. Electromagnetic Constraint

Let us image a BH as a gas of particles packed in a small region by the gravitational force (The model of BH I will consider may remind of the one discussed in [15]. The radius of the compact object, *R*, is larger than the one corresponding to the event horizon of a (classical) BH with the same mass and spin.). As this gas has a finite temperature, it must radiate. However, if the object is very compact, the emitted radiation is strongly redshifted when it reaches a distant observer and the object can appear very faint. Here, I relax the quite common assumption of steady state $L=\dot{M}c^{2}$ [9, 10, 12, 13], where *L* is the surface luminosity and $\dot{M}$ is the mass accretion rate. That would require that the accreting gas hits the “solid surface” of the object and then radiates to infinity all its kinetic energy. If this were the case, a very compact object would not be able to increase its mass, or at least the process would be very inefficient, likely in contradiction with the observations of the supermassive objects in galactic nuclei. Moreover, there are no reasons to assume that BH candidates have a solid surface. In the picture in which we have a gas of particles packed in a small region by the gravitational force, the accreting gas enters into the compact object and both its rest-mass and kinetic energy contribute to increasing the mass of the BH candidate.

Let us now see the constraint we can obtain in this picture from the nonobservation of thermal spectrum from BH candidates. The specific energy flux density of the compact object (often measured in erg cm^−2^ s^−1^ Hz^−1^) as detected by a distant observer is as follows:

(2) $$\begin{array}{c} F=\int I_{o}d\Omega , \end{array}$$

where *I*_*o*_ is the specific intensity of the radiation as measured by the distant observer and *dΩ* is the element of the solid angle subtended by the image of the object on the observer's sky. *I*_*x*_/*ν*_*x*_^3^ = const. (Liouville's Theorem), where *ν*_*x*_ is the photon frequency measured by any local observer on the photon path, and

(3) $$\begin{array}{c} d\Omega =\frac{dx\;dy}{D^{2}}, \end{array}$$

where *x* and *y* are the Cartesian coordinates on the observer's sky and *D* is the distance of the compact object from the observer. The *equivalent isotropic luminosity* of the BH candidate is thus

(4) $$\begin{array}{c} L=4\pi \int g^{3}I_{e}\;dx\;dy\;d\nu . \end{array}$$

Here, *g* = *ν*_*o*_/*ν*_*e*_ is the redshift factor, *ν*_*o*_ is the photon frequency measured by the distant observer, and *ν*_*e*_ and *I*_*e*_ are, respectively, the photon frequency and the specific intensity of the radiation measured by an observer located at the point of emission of the photon, on the surface of the compact object, and corotating with the surface of the compact object. The emission should be like the one of a blackbody; that is,

(5) $$\begin{array}{c} I_{e}=\frac{2h\nu_{e}^{3}}{c^{2}}\frac{1}{\exp\left(h\nu_{e}/k_{B}T_{e}\right)-1}, \end{array}$$

where *T*_*e*_ is the temperature of the surface of the BH candidate measured by a locally corotating observer.

For the sake of simplicity, we now consider a spherically-symmetric nonrotating object. The geometry of the spacetime around the BH candidate will be described by the Schwarzschild solution, which is valid till the radius of the compact object, *R*. The luminosity becomes as follows:

(6) $$\begin{array}{c} L=4\sigma g^{4}T_{e}^{4}\int dx\;dy, \end{array}$$

where *σ* is the Stefan-Boltzmann constant and

(7) $$\begin{array}{c} g={\left(1-\frac{2M}{R}\right)}^{1/2}. \end{array}$$

Here, *g* is a constant, but it would be a function of *x* and *y* in a more general background. The integrand in (6) is simply the area of the apparent image of the BH candidate on the observer's sky:

(8) $$\begin{array}{c} \int dx\;dy=\pi R_{\text{app}}^{2}=\{\begin{array}{cc} 27\pi M^{2} & R<3M\\ \pi \frac{R^{2}}{g^{2}} & R>3M. \end{array} \end{array}$$

The radius *r* = 3*M* is the capture photon radius of the Schwarzschild spacetime. Inside such a radius, the gravitational force is so strong that any light ray coming from infinity is captured by the compact object.

A distant observer sees therefore an object with an apparent temperature

(9) $$\begin{array}{c} T_{\text{app}}=gT_{e}\approx T_{e}{\left(\frac{\delta }{2M}\right)}^{1/2}, \end{array}$$

where I wrote *R* = 2*M* + *δ* and assumed *δ* ≪ 1 and positive. The most stringent constraint on *δ* can be inferred from the observations of the supermassive BH candidate at the center of our Galaxy. Infrared and near-infrared data require *T*_app_ < 0.01 eV [12, 13]. If we assume a local temperature as high as *k*_*B*_ *T*_*e*_ ~ *m*_*p*_ *c* ^2^ ~ 1 GeV (roughly the gravitational binding energy of a proton), we find the following:

(10) $$\begin{array}{c} \delta <10^{-10}\;\text{cm}, \end{array}$$

as *M* ≈ 6 · 10^11^ cm. With a lower temperature *T*_*e*_, the constraint would be weaker, while a higher temperature seems to be unlikely, as the object is old and the accreting gas would have already cooled it down. The proper distance of the boundary of the BH candidate from the event horizon of a Schwarzschild BH with the same mass is as follows:

(11) $$\begin{array}{c} \Delta \approx \frac{\delta }{\sqrt{1-\left(2M/R\right)}}\approx \sqrt{2M\delta }<10\;\text{cm}. \end{array}$$

Such a result should not change significantly if we consider a rotating object.

## 3. Stability Constraint

The existence of event or apparent horizons in astrophysical BH candidates is also suggested by considerations concerning the stability of these objects. It is well known that rapidly rotating very compact objects may be affected by the *ergoregion instability* [16–19]. In the ergoregion, *g*_*tt*_ > 0 (if the metric has signature −+++) and the frame dragging is so strong that stationary orbits are not allowed. That implies that in the ergoregion there are excitations with negative energy with respect to a stationary observer at infinity. These excitations can be seen as quasi-bound states: they are trapped by the gravitational potential on the one side, and by the surface of the object (or by the center of the object if the latter is made of matter noninteracting with the excitations) on the other side. As some modes can escape to infinity carrying positive energy, negative energy modes in the ergoregion can grow indefinitely, thus generating an instability. Objects with a horizon may instead be stable because there may not be quasi-bound states in the ergoregion: any excitation in the ergoregion is swallowed by the BH. Let us notice, however, that the existence of a horizon is not sufficient in general to prevent the ergoregion instability [20].

Roughly speaking, the instability timescale *τ* decreases as the angular velocity and the compactness of the compact object increase. For rotating very compact objects, one typically finds that the instability is strong and occurs on a dynamical timescale *τ* ~ *M* [21]; that is, ~1 s for objects with a mass *M* ~ 10*M*_⊙_ and ~10^7^ s if *M* ~ 10^8^ *M*_⊙_. While there are counterexamples in which rotating compact objects can be stable or very long living [22], it seems difficult that the latter can still meet observations requiring that astrophysical BH candidates can rotate very rapidly [23–27] and have a high radiative efficiency [28–31]. Let us notice, however, that the issue of the ergoregion instability can be discussed only within a well-defined theoretical model (gravity theory, internal structure, and composition of the compact object, etc.) and that it has been studied only for a very limited number of specific cases. Considerations on the nonobservations of electromagnetic radiation from the surface of BH candidates are much more model independent and rely on a set of assumptions that can be violated only invoking very exotic new physics.

Here, I will discuss the ergoregion instability within the following picture. I assume that the geometry around an astrophysical BH candidate is exactly described by the Kerr solution up the radius of the compact object, *R*. Considerations on the ergoregion instability indeed require a specific background and we may think that possible deviations from the Kerr metric can be tested with other approaches [32]. In the case of a reflecting surface, the timescale for scalar instabilities can be estimated as follows [33]:

(12) $$\begin{array}{c} \tau \sim A\left(M,a_{*}\right)\left|\ln\left(\frac{R-R_{H}}{2M\sqrt{1-a_{*}^{2}}}\right)\right|, \end{array}$$

where $R_{H}=M(1+\sqrt{1-a_{*}^{2}})$ is the radius of the event horizon of a Kerr BH with mass *M* and spin parameter *a*_∗_. *A*(*M*, *a*_∗_) is a function of *M* and *a*_∗_. For moderate values of the spin parameter *a*_∗_, *A* ~ *M*; that is, the instability occurs on a dynamical timescale. For high values of *a*_∗_, *A* decreases very quickly. In the case of a Kerr BH, *R* = *R*_*H*_ and the object is stable. On the other hand, if *R* = *R*_*H*_ + *δ*, the fact that we observe long-living rapidly rotating BH candidates demands

(13) $$\begin{array}{c} \delta ,\Delta \ll L_{\text{Pl}}\approx 10^{-33}\;\text{cm}, \end{array}$$

where Δ is the physical distance encountered in the previous section. Equation (13) essentially rules out the possibility that current BH candidates have no horizon, or at least something that behaves very much like a horizon for the unstable modes. The possibility of an exact Kerr background with *δ* so large that there is no ergoregion seems to be unlikely, as we know objects that, when the spacetime around them is described by the Kerr solution, would have an accretion disk with inner edge inside the ergosphere [23–25].

## 4. Conclusions

In conclusion, we have observations suggesting that BH candidates have a horizon or at least putting constraints on the possible distance between the boundary of these compact objects and the event horizon of a BH with the same mass and spin. Such a distance can be seen as a measurement of how much close the formation of the horizon is. From the nonobservation of thermal radiation from the putative surface of astrophysical BH candidates, one can infer the constraint in (10) and (11): actually, such a bound is not so stringent, as one may argue that new physics can show up at much shorter scales. However, the result seems to be quite robust—it is just supposed that the compact object must emit electromagnetic radiation due to its finite temperature—and very exotic new physics is necessary to change these conclusions or to get a different bound. Considerations on the ergoregion instability are instead to be taken with caution. The timescale instability strongly depends on the exact model, that is, gravity theory, internal structure, and composition of the object, and so on, which we do not know. However, we can optimistically arrive at the following conclusion. If the geometry around astrophysical BH candidates is very close to the Kerr solution, the existence of stable or long-living objects likely requires some kind of horizon. Otherwise, we can probably hope to discover deviations from the Kerr background with tests already proposed in the literature and possible in a near future with new observational facilities.
